# Trends and Socio-Demographic Differences of Cannabis Vaping in the USA and Canada

**DOI:** 10.3390/ijerph192114394

**Published:** 2022-11-03

**Authors:** Carmen C. W. Lim, Gary C. K. Chan, Elle Wadsworth, Daniel Stjepanović, Vivian Chiu, Jack Y. C. Chung, Tianze Sun, Jason Connor, Janni Leung, Coral Gartner, Wayne Hall, David Hammond

**Affiliations:** 1 National Centre for Youth Substance Use Research, Faculty of Health and Behavioural Sciences, The University of Queensland, St Lucia, QLD 4067, Australia; 2 School of Psychology, Faculty of Health and Behavioural Sciences, The University of Queensland, St Lucia, QLD 4067, Australia; 3 School of Public Health Sciences, University of Waterloo, 200 University Ave W, Waterloo, ON N2L 3G1, Canada; 4 Canadian Centre on Substance Use and Addiction, 75 Albert St, Suite 500, Ottawa, ON K1P 5E7, Canada; 5 Discipline of Psychiatry, Faculty of Medicine, The University of Queensland, Herston, QLD 4006, Australia; 6 NHMRC Centre of Research Excellence on Achieving the Tobacco Endgame, School of Public Health, Faculty of Medicine, The University of Queensland, Herston, QLD 4006, Australia; 7 Queensland Alliance for Environmental Health Sciences, The University of Queensland, Woolloongabba, QLD 4102, Australia

**Keywords:** cannabis vaping, marijuana vaping, cannabis

## Abstract

Given the rise in cannabis vaping, it is important to highlight the heterogeneity in vaping different cannabis product because of the potential differences in their health risks. This study aims to estimate the trends and socio-demographic correlates of the use of various cannabis vaping products across jurisdiction with different legal status. Data from the 2018 (n = 27,169) and 2019 (n = 47,747) waves of the International Cannabis Policy Study (ICPS) were used. Respondents aged 16–65 completed web-based surveys. In 2019, proportions of past year vaping of cannabis oil, dried flower and concentrates in the overall sample were highest in U.S. jurisdictions where cannabis was legalized for non-medical use (17.4%, 6.0%, 4.9%), followed by U.S. jurisdiction where non-medical cannabis use is illegal (13.7%, 5.8%, 2.9%), and lowest in Canada (8.1%, 4.4%, 2.1%). Vaping dried flower decreased from 2019 to 2018 in U.S. legal jurisdictions and Canada, while vaping cannabis oil and concentrates increased in all jurisdictions (*p* < 0.001). The odds of vaping all forms of products were higher among younger respondents (16–55 years), males, respondents with some college education, and persons with low-risk perceptions on daily cannabis vaping. In both ICPS surveys (2018 and 2019), cannabis oil was the most frequently vaped products, followed by dried flower, and concentrates. Detailed measures of product forms for cannabis vaping should be considered in future surveys.

## 1. Introduction

As of 2022, Canada and the United States (U.S.) have expansive cannabis markets that provide a wide range of cannabis products. Although dried flower was the predominant product used in both countries, the use of other product forms has increased between 2017 and 2020 [1,2]. Vaping is a popular mode of administration by heating cannabis products to release its active psychoactive compounds into vapor for inhalation [3]. The design of vaporizers has evolved to reflect the changing preferences of cannabis consumers. The earliest marketed vaporizer designed for dried flower was a portable, battery-powered desktop vaporizer [4]. Vaping cannabis oil using disposable or refillable cartridges has emerged more recently, in parallel with product advancements for nicotine containing ‘e-cigarettes’ [5]. Vaping devices have also been developed to facilitate vaping of ‘solid’ concentrates such as wax or shatter in which the concentrate is placed in a chamber or on a metal component that heats it, but below the point of combustion [5].

Multiple reviews and studies found an increasing prevalence of cannabis vaping among the general population in the U.S. and Canada in the past decade [6,7,8,9]. A 2018 nationally representative survey of 131,807 U.S. adults found that 1.3% reported vaping cannabis in the past month and, among people who used nicotine ‘e-cigarettes’, 7.1% reported vaping cannabis in the past month [7]. Three recent reviews reported an increasing number of young people vaped cannabis using a personal vaporizer or e-cigarettes between 2013 to 2021 in the U.S. and Canada [6,8,9]. A meta-analysis of 17 national studies showed the past month pooled prevalence of cannabis vaping has increased by 5-fold from 1.6% in 2013–2016 to 8.4% in 2019–2020 [6].

Given the rapid growth of cannabis vaping, nationally representative surveys may underestimate the prevalence and population-related harms by measuring cannabis vaping with a single question. For example, the 2018 National Youth Tobacco Survey in the U.S. used the following question “Have you ever used marijuana, marijuana concentrates, marijuana waxes, THC (Δ9-tetrahydrocannabinol), or hash oils in an e-cigarette?” A few surveys have separated this question into product type (e.g., vaping dried flower, cannabis oil, or extracts) but they were based on small, non-representative of respondents using cannabis and/or e-cigarettes [10,11,12,13,14]. These studies showed the lifetime and/or past month rates of vaping dried flower and cannabis concentrates were different. For example, Morean and colleagues reported that 45.5%, 15.2%, and 39.4% of the respondents who have vaped cannabis indicated that their preferred vaping products were hash oil, THC wax, and dried flower, respectively [13]. There is a need for replicating these estimates using a larger sample of respondents.

Vaping different cannabis products may pose different health risks. Vaping dried flower for example, generates fewer toxicants compared to smoking dried flower [3], while producing a similar pharmacokinetic profile as smoking in terms of the onset, intensity, and duration of psychoactive effects [15]. However, it is debatable whether vaporizing cannabis is lower risk than smoking cannabis because the lower toxicity of cannabis vaping needs to be weighed against the increased risk of behavioral, psychological, and neurocognitive effects of high THC content products [3]. For example, some highly potent vaping products in the form of oil and concentrates can have THC levels up to 70% [16], compared to dried flower products, which generally do not contain more than 30% THC [17,18,19]. Regular use of highly potent products is associated with cognitive impairment, dependence syndrome, elevated risk of psychosis, and more severe cannabis withdrawal [20,21,22,23]. 

Existing research have focused on the socio-demographic characteristics of youth and young adults who vape cannabis [9]. Less is known about the socio-demographic characteristics of older adults (>35 years) who vape cannabis. A few studies that analyzed the socio-demographic characteristics of adults who vaped cannabis have either examined a limited set of correlates [24], have included socio-demographics as covariates in their analysis [25], or have categorized adults above 35 as a single category [26]. For example, Mattingly and colleagues limited the age group comparison to young adults (age 18–24, age 25–34) versus older adults (35+)[26]. It is important to draw the distinction especially with the older adult population because people in this age-group also demonstrated an increase in cannabis use to relieve symptoms of chronic medical conditions [27].

In addition, cannabis laws in the U.S. and Canada have changed from 2018 to 2019: non-medical cannabis was legal in 9 U.S. jurisdictions in 2018 and across 10 U.S. jurisdictions and in the District of Columbia (D.C) in 2019, while Canada legalized sales of dried flower and some oils at the end of 2018 and all other products (e.g., extracts) in December 2019. Cannabis extracts, edibles and topicals were not available for legal sale in Canada until January 2020 onwards. The legalization of non-medical cannabis use is likely to increase the accessibility and the affordability of potent cannabis products [23,28]. Daniulaityte and colleagues [10] showed those who vaped concentrates were more likely to live in a jurisdiction where non-medical cannabis use is legal and have a lower perceived risk of cannabis use. Another study found jurisdictions with a longer duration of legal cannabis laws (non-medical or medical cannabis law) and areas with a higher density of dispensaries were associated with an increased odds of vaping cannabis [29]. 

This study aims to use data from large samples from the International Cannabis Policy Study to estimate the past year proportion of vaping dried flower, cannabis oil or liquid, and concentrates (i) by jurisdiction type (Canada and U.S. jurisdictions where cannabis is and is not legal for non-medical use), (ii) by survey year (2018 vs. 2019), and (iii) to assess its socio-demographic correlates. 

## 2. Materials and Methods

### 2.1. Setting

Data are from the 2018 and 2019 International Cannabis Policy Study (ICPS) [30] with sample sizes of 27,169 and 47,747 respectively. Individuals were eligible to participate if they resided in a Canadian province or U.S. state, were 16–65 years of age at the time of recruitment with Internet access. A non-probability sample of respondents was recruited through the Nielsen Consumer Insights Panel and their partners’ panels. The Nielsen panels were recruited using a mixture of probability and non-probability sampling methodology. Nielsen drew stratified random samples from the online panels in each country, based on known proportions in each age group in each jurisdiction. To account for differential response rates, Nelsen modified these sampling proportions to place greater weight on sub-group with lower response rates. Email invitations with a unique link were sent to a random sample of panelists after targeting for age and country criteria; ineligible panels were not invited. The web-based survey was conducted in the English language, which was also available in French as an additional option for respondents in Canada. Informed consent was obtained, and each respondent received remuneration according to their panel’s usual incentive structure. The completion rates for the 2018 and 2019 surveys were 64.2% and 62.9% respectively. A small sample of 2019 ICPS respondents (n = 2012, 7.4%) have participated in the 2018 ICPS study. Due to the small overlap, it is more appropriate to analyze this as two cross-sectional surveys. Post-stratification weights were constructed to reflect the age, sex, race/ethnicity distribution of the respondents in each jurisdiction. Cross-sectional findings of both surveys are presented in this study. U.S. legal jurisdictions included respondents in jurisdictions where non-medical cannabis laws were implemented at the time of data collection (i) in August–October 2018 (Alaska, California, Colorado, Maine, Massachusetts, Nevada, Oregon, Vermont, and Washington) and (ii) in September 2019 (Alaska, California, Colorado, Maine, Massachusetts, Michigan, Nevada, Oregon, Washington, Vermont, and District of Columbia). U.S. illegal jurisdictions include respondents where non-medical cannabis was prohibited at the time of data collection. 

### 2.2. Measures

Three cannabis vaping variables were created: (i) vaping dried flower, (ii) vaping cannabis concentrates, and (iii) vaping cannabis oil. Respondents were asked if in the last year “Have you used marijuana in any of the following ways?” Options to this question include “Dried herb (smoked or vaped)”, “Cannabis oils or liquids for vaping”, “Concentrates (e.g., wax, shatter, budder)”. In the ICPS, because dried flower and concentrates can be smoked or vaped, the survey questions to measure their use differed from those for cannabis oil or liquids. “Respondents were further asked, “Of all the dried herb that you used in the past 12 months, what percent (%) do you… vape (%)”. Vaping dried flower was coded as positive if respondents reported using at least 1% of the dried flower they used was vaped. The same coding was used for vaping concentrates (positive if at least 1% of concentrates vaped in past 12 months). Vaping cannabis oil was coded as positive if the respondents endorse a “yes” for having vaped cannabis oil or liquids.

The following variables were used in the analysis: sex (‘females’, ‘males’), age-group (‘16–25’, ‘26–35’, ‘36–45’, ‘46–55’, ‘56–65’), education level (‘less than high school’, ‘high school diploma or equivalent’, ‘some college or equivalent’, ‘bachelor’s degree or higher’), ethnicity (‘white’, ‘other’), perception on the risks of daily cannabis vaping (‘low risk’, ‘moderate risk’, ‘high risk’). Total personal income (before taxes) over the past 12-months was coded by dividing the income distribution into quartiles (“low-income”, “low-average income”, “high-average income”, “high income”). Perception on the risks on the risk of daily cannabis vaping was based on the question “In your opinion, what is the level of health risk from vaping marijuana daily”.

### 2.3. Analysis

The proportion of past year cannabis vaping by product type was estimated for each jurisdiction. Univariable comparison was performed to compare the differences between 2018 and 2019 for each type of product. Multivariable logistic regression was used to estimate the associations between socio-demographics and the three cannabis vaping outcomes (vaping dried flower, cannabis concentrates, and cannabis oil or liquid). Missing values for correlates range from 0.5% (education level) to 15.2% (risk perception). Multiple imputation by chained equations (20 imputations) were performed. All analyses were weighted to match the samples to the population sociodemographic distribution within each jurisdiction. Significance level, α, was set at 0.00208 (α = 0.05/24) to adjust for multiple comparisons. This approach was based on the Bonferroni correction which is often deemed to be too conservative. Another approach is to set the threshold of the alpha level to 0.01. Regardless of the approach used, the results remained unchanged, and we choose to retain the former approach. Analyses were performed using STATA version 17. 

## 3. Results

### 3.1. Sample Characteristics

The sample characteristics of the ICPS samples are shown in Appendix A. There was an approximately equal split of respondents in each age group (age range 16 to 55) and sex. Over a quarter had Bachelor’s degree or higher education (2018 range = 24.7% in Canada to 29.9% in U.S. legal jurisdictions; 2019 range = 24.6% in Canada to 32.5% in U.S. legal jurisdictions). Around 23% to 27% of the respondents identified as ‘white’. Approximately half of the participants perceived daily cannabis vaping as a high-risk activity (2018 range = 41.2% in U.S. illegal jurisdictions to 51.4% in Canada; 2019 range = 51.2% in U.S. illegal jurisdictions to 59.3% in Canada).

### 3.2. Past Year Proportion of Cannabis Vaping

Table 1 shows the proportion of past year cannabis vaping by product type across Canada, U.S. legal jurisdictions, and U.S. illegal jurisdictions. For country comparison, the past year proportion of all forms of vaping (i.e., vaping dried flower, cannabis oil or liquid, or concentrates) was highest in U.S. legal jurisdictions, followed by U.S. illegal jurisdictions and Canada. For example, in 2019, vaping dried flower was the highest in U.S. legal jurisdictions (6.0%, 95% C.I. = 5.6–6.5%) and the lowest in Canada (4.4%, 95% C.I. = 4.0–4.8%). Cannabis oil or liquid was found to be the most common vaping product in each jurisdiction, followed by dried flower and concentrates. Vaping dried flower decreased from 2019 to 2018 in U.S. legal jurisdictions and Canada, while vaping cannabis oil and concentrates increased in all jurisdictions (*p* < 0.001).

### 3.3. Socio-Demographic Correlates of Past Year Cannabis Vaping

Table 2 shows the multivariable logistic regression model between socio-demographic correlates and three cannabis vaping outcomes. Socio-demographic correlates of cannabis vaping by jurisdiction type can be found in Appendix B–D. Overall, those in younger age groups, especially those aged (16–25 years), males, those with some college or equivalent education, and those that perceived daily cannabis vaping as a low or moderate risk activity were at increased odds of vaping all forms of cannabis products (dried flower, cannabis oil or liquid, and concentrates). Having a high school diploma or equivalent education was associated with vaping of any forms of cannabis product (OR = 1.3 [1.0–1.6]). Ethnicity was only associated with vaping concentrates when respondents identified being ‘white’ (OR = 1.3 [1.0–1.6]). Respondents with high-average (OR = 1.4 [1.1–1.8]) or high (OR = 1.6 [1.2–2.0]) personal income when compared with low personal income were associated with increased odds of vaping dried flower but not other forms of cannabis products.

## 4. Discussion

Based on 74,916 ICPS respondents from the U.S. and Canada, the proportion of past year cannabis vaping differed by type of cannabis product vaped. Across all jurisdictions, cannabis oil was the most frequently vaped product, followed by dried flower, and concentrates. For example, in 2019, around 8.1 to 17.4% of respondents were vaping cannabis oil whereas the proportions vaping of dried flower and concentrates were 2–4 times lower than that. As legalization continues in North America, the diversification of cannabis products and novel delivery method highlights the importance of considering separate questions for each product type (dried flower, oil, or solid concentrates) for vaping in future national surveys. 

All forms of vaping were found to be the higher in U.S. legal jurisdictions than U.S. illegal jurisdictions and Canada. This is consistent with cannabis legalization increasing accessibility to vaping products [23]. Legal cannabis market can implement product standards to minimize any excess risks due to contaminants or poor manufacturing, but they may accelerate the uptake of vaping highly manufactured cannabis extracts (THC or CBD rich). Our study also showed all forms of vaping was more common in U.S. illegal jurisdictions compared to Canada. It is important to note that at the time of data collection in 2018, cannabis was not yet legal in Canada. Canada legalized cannabis for non-medical use in October 2018 but Canadian adults were only able to access cannabis extracts and edibles a year later (i.e., October 2019). The widespread availability of highly potent products from licit sources (clearnet [31]) and retail stores [32]) and the darknet [33] suggests a need to monitor the changing profile and nature of cannabis vaping at a population level. 

The prevalences of vaping cannabis oil and concentrates were higher in 2019 than 2018 in all jurisdictions. Conversely, vaping dried flower decreased by 3.6% in U.S. legal jurisdictions and by 0.3% in Canada in 2019 but not in U.S. illegal jurisdictions. Early sales data from Washington’s legal non-medical market found a shift in the market share from dried flower towards cannabis extracts [34]. Evidence on the impact of the shift in consumption from dried flower to newer product forms remains scarce. It was reported that more people were likely to try new cannabis products after cannabis was legalized for non-medical use in the state of Colorado which led to symptoms of paranoia and hallucination, and presentation to emergency rooms [35]. In Canada, there was also an increase in cannabis-related emergency department visits after cannabis extracts and edibles were permitted for sale in Canada [36].

Lower risk cannabis use guidelines have recommended the use of ‘low potency’ cannabis products and of vaping rather than smoking dried flower as a harm minimization approach [37]. These recommendations would imply avoiding vaporizing cannabis extracts and oils with high levels of THC. It remains unclear whether consumers titrate their THC intake when they are using higher versus lower THC products [19,38]. A better understanding of cannabinoid content and concentrations is a priority for future research. Future state governments may consider adopting a potency-based tax to reduce the externalities of cannabis consumption. 

This study also found younger age-groups were likely to vape any form of cannabis. The International Tobacco Control Policy Evaluation Project (ITC) Youth Tobacco and Vaping Survey examined changes in modes of cannabis consumption among young people in England, Canada, and the U.S. The study found the use of e-cigarettes to vape cannabis oil or liquid increased by more than 2-fold from 2017 to 2019 while smoking cannabis (with tobacco in England and without tobacco in the U.S.) decreased [2]. This movement toward vaping to administer cannabis also parallels the youth e-cigarette use literature, which has seen nicotine vaping overtake smoking tobacco among US youth [39]. It may be this common route of administration through inhalation of vaping products that has led young people who use cannabis to opt for vaping rather than smoking it. However, while the overall youth nicotine product use has been variable, trending up from 2017 to 2019 before trending down again between 2019 and 2021[40], the overall prevalence of cannabis use in young people from multiple countries has remained relatively stable even after medical and/or non-medical cannabis legalization [41,42]. Future research should collect information by delivery method and types of product use to inform preventative efforts. This is especially important given the rapid increase in the uptake of cannabis vaping in this age group [6] if we are to identify ways to reduce cannabis-related harms in this population.

In this study, all forms of cannabis vaping were associated with those who perceived daily cannabis vaping to be a low to moderate risk activity. A study based on the U.S. National Survey on Drug Use and Health found a doubling in the prevalence of persons who perceived cannabis use to be low risk between 2002 (17%) and 2018 (36%) [43]. This has implications as those who perceived cannabis use as low risk were 6-times more likely to report cannabis use in the past year than those who perceived cannabis use as high risk [43]. Another study found a positive link between perception of cannabis use as a low-risk activity was linked to driving under the influence of cannabis [44]. Future studies should continue to monitor the perceptions of cannabis vaping given the rapid change in the accessibility and affordability of cannabis products.

### Limitations

Although the ICPS study provides a unique opportunity to directly compare the proportion of cannabis vaping across jurisdictions using a common survey instrument, this study is not without its limitations. The ICPS study is based on self-report and under-reporting due to stigma and legality may underestimate actual proportion of use. Respondents were recruited using non-probability-based sampling; therefore, the findings do not necessarily provide nationally representative estimates. However, the data in our study were weighted to reflect the actual population structure in both the U.S. and Canada. Cannabis use estimates in the ICPS were within the range of national estimates for young adults, whereas estimates among the full ICPS sample were generally higher than national surveys in the U.S. and Canada [30]. This is likely because the ICPS sampled individuals aged 16–65, whereas the national surveys included older adults, who have lower rates of cannabis use. All the U.S. jurisdictions were grouped as legal or illegal jurisdictions, which could suppress heterogeneities in cannabis laws across individual jurisdictions. Finally, the current study was unable to evaluate the cannabinoid content (THC and CBD levels) of cannabis products because of a high proportion of missing values.

## 5. Conclusions

The proportion of ICPS respondents vaping cannabis increased in Canada and the United States between 2018 and 2019, largely driven by vaping of THC oils or liquids. Vaping ‘solid’ concentrates increased only to a modest extent, whereas vaping dried flower decreased between 2018 and 2019 in U.S. legal jurisdictions and Canada. The data suggest an overall increase in cannabis vaping and a shift towards vaping of higher THC content products, particularly in U.S. states that have legalized non-medical cannabis use. In addition, vaping cannabis was considerably more common among youth and young adults. The findings highlight the rapidly evolving cannabis market and underscore the importance of collecting more detailed measures of cannabis consumption that can account for different modes of delivery, product forms and cannabinoid content.

## Figures and Tables

**Table 1:** Past year proportion of cannabis vaping, by product and jurisdiction.

	Vaping Dried Flower ^a^				Vaping Oil or Liquid ^b^				Vaping Concentrates ^c^				Any Vaping ^d,e^			
	n	%	95% C.I.		n	%	95% C.I.		n	%	95% C.I.		n	%	95% C.I.	
**2018**																
Canada (n = 10057)	430	5.1	4.5	5.7	459	5.8	5.1	6.4	146	1.8	1.4	2.2	710	8.4	7.6	9.2
U.S. illegal (n = 9714)	426	5.5	4.9	6.1	579	7.2	6.5	7.9	146	2.0	1.6	2.4	746	9.6	8.8	10.4
U.S. legal (n = 7398)	541	9.6	8.4	10.8	813	13.6	12.3	15.0	214	3.8	3.0	4.5	984	16.4	14.9	17.9
**2019**																
Canada (n = 16285)	620	4.4	4.0	4.8	1094	8.1	7.6	8.7	285	2.1	1.8	2.4	1440	10.4	9.8	11.0
U.S. illegal (n = 10433)	540	5.8	5.3	6.4	1412	13.7	12.9	14.5	277	2.9	2.5	3.4	1561	15.4	14.6	16.2
U.S. legal (n = 21029)	1018	6.0	5.6	6.5	3350	17.4	16.7	18.1	858	4.9	4.5	5.3	3698	19.6	18.9	20.3

^a^ Significance difference between 2018 and 2019 (t = −4.12, *p* < 0.001). ^b^ Significance difference between 2018 and 2019, all jurisdictions combined (t = 13.3, *p* < 0.001). ^c^ Significance difference between 2018 and 2019, all jurisdictions combined (t = 4.2, *p* < 0.001). ^d^ Any vaping is a combination of vaping dried flower, cannabis oil or liquid or concentrates. ^e^ Significance difference between 2018 and 2019, all jurisdictions combined (t = 11.2, *p* < 0.001).

**Table 2:** Socio-demographics correlates and risk perception of vaping dried flower, oil or liquid, and concentrates in the past year, all jurisdictions ^a^.

	Vaping Dried Flower				Vaping Oil or Liquid				Vaping Concentrates				Any Vaping			
	aOR	99.79% C.I		[*p*]	aOR	99.79% C.I		[*p*]	aOR	99.79% C.I		[*p*]	aOR	99.79% C.I		[*p*]
**Jurisdictions (ref: Canada)**																
U.S. illegal	1.0	0.8	1.2	0.847	1.3 *	1.1	1.5	<0.001	1.1	0.8	1.4	0.464	1.1 *	1.0	1.3	0.001
U.S. legal	1.3 *	1.1	1.6	<0.001	2.1 *	1.8	2.4	<0.001	1.9 *	1.4	2.4	<0.001	1.8 *	1.6	2.0	<0.001
**Age-group (ref: 56–65 years)**																
16–25 years	4.3 *	3.2	5.7	<0.001	3.3 *	2.7	4.0	<0.001	4.7 *	3.1	7.1	<0.001	3.6 *	3.0	4.3	<0.001
26–35 years	3.8 *	2.9	4.9	<0.001	3.4 *	2.8	4.0	<0.001	4.3 *	2.9	6.3	<0.001	3.5 *	3.0	4.1	<0.001
36–45 years	2.7 *	2.0	3.5	<0.001	2.6 *	2.2	3.1	<0.001	3.5 *	2.4	5.3	<0.001	2.6 *	2.2	3.1	<0.001
46–55 years	1.6 *	1.2	2.2	<0.001	1.6 *	1.4	2.0	<0.001	1.9 *	1.2	2.9	<0.001	1.6 *	1.4	1.9	<0.001
**Sex (ref: female)**																
Male	1.5 *	1.3	1.8	<0.001	1.3 *	1.1	1.4	<0.001	1.6 *	1.4	2.0	<0.001	1.3 *	1.2	1.5	<0.001
**Education level (ref: less than high school)**																
High school diploma or equivalent	1.1	0.8	1.5	0.199	1.2	1.0	1.5	0.012	1.4	0.9	2.1	0.017	1.3 *	1.0	1.6	<0.001
Some college or equivalent	1.4 *	1.0	1.8	0.001	1.4 *	1.1	1.7	<0.001	1.6 *	1.1	2.5	<0.001	1.5 *	1.2	1.8	<0.001
Bachelor’s degree or higher	1.3	1.0	1.8	0.005	1.1	0.9	1.3	0.329	1.3	0.8	1.9	0.114	1.2	1.0	1.5	0.006
**Ethnicity (ref: other)**																
White	1.0	0.9	1.2	0.611	1.1	1.0	1.2	0.016	1.3 *	1.0	1.6	0.001	1.1	1.0	1.2	0.011
**Total personal income (ref: low)**																
Low-average	1.2	1.0	1.5	0.005	1.1	1.0	1.3	0.017	1.0	0.8	1.4	0.642	1.2	1.0	1.3	0.002
High-average	1.4 *	1.1	1.8	<0.001	1.1	0.9	1.3	0.037	1.0	0.8	1.4	0.661	1.2 *	1.0	1.4	0.001
High	1.6 *	1.2	2.0	<0.001	1.1	0.9	1.3	0.045	1.3	0.9	1.7	0.032	1.2 *	1.0	1.4	0.001
**Perception on the risk of daily cannabis vaping (ref: high risk)**																
Low risk	5.6 *	4.6	6.7	<0.001	7.0 *	6.1	8.0	<0.001	6.7 *	5.2	8.6	<0.001	7.1 *	6.3	8.1	<0.001
Moderate risk	3.0 *	2.4	3.6	<0.001	3.6 *	3.2	4.2	<0.001	3.3 *	2.5	4.3	<0.001	3.5 *	3.1	4.0	<0.001

^a^ Each multivariable logistic regression model included survey year, jurisdiction, age-group, sex, education level, ethnicity, total personal income, and perception on the risk of daily cannabis vaping as covariates. * aORs (adjusted odds ratios) were significant at the 0.002 level.

## Data Availability

Details about the ICPS project and access to ICPS dataset can be found here: http://cannabisproject.ca/methods/ (accessed on 30 October 2022).

## Appendix A

**Table A1:** Socio-demographics correlates and risk perception of cannabis vaping by survey year and jurisdiction type.

	2018 (n = 27,169)									2019 (n = 47,747)								
	Canada			U.S. Illegal Jurisdictions			U.S. Legal Jurisdictions			Canada			U.S. Illegal Jurisdictions			U.S. Legal Jurisdictions		
	%	95% C.I.		%	95% C.I.		%	95% C.I.		%	95% C.I.		%	95% C.I.		%	95% C.I.	
**Age-group**																		
16–25 years	18.9	17.9	20.0	19.9	19.1	20.8	19.6	17.9	21.2	18.8	18.0	19.6	19.9	19.0	20.7	19.8	19.0	20.6
26–35 years	20.7	19.5	22.0	21.4	20.2	22.6	23.0	21.4	24.6	20.9	20.1	21.7	21.5	20.6	22.5	22.7	21.9	23.4
36–45 years	19.6	18.4	20.7	18.9	17.9	20.0	17.3	15.8	18.8	19.8	19.0	20.5	19.1	18.2	19.9	19.4	18.7	20.0
46–55 years	20.8	19.7	21.9	20.1	19.2	21.1	21.7	20.1	23.4	19.9	19.2	20.7	19.8	18.9	20.8	19.4	18.7	20.1
56–65 years	20.0	19.1	20.9	19.6	18.8	20.3	18.4	17.1	19.7	20.6	19.9	21.4	19.7	18.9	20.5	18.8	18.2	19.4
**Sex**																		
Males	50.2	48.8	51.5	49.7	48.4	50.9	50.3	47.8	51.6	50.3	49.3	51.3	49.7	48.6	50.8	50.2	49.4	51.1
Females	49.8	48.4	51.2	50.3	49.1	51.5	49.7	47.8	51.6	49.7	48.7	50.7	50.3	49.2	51.4	49.8	48.9	50.6
**Education level**																		
Less than high school	15.4	14.3	16.5	15.2	14.4	16.0	11.8	10.3	13.2	15.4	14.6	16.3	12.1	11.4	12.8	5.1	4.6	5.5
High school diploma or equivalent	26.6	25.2	27.9	19.4	18.4	20.5	15.9	14.5	17.3	26.4	25.5	27.4	22.5	21.5	23.5	20.3	19.5	21.0
Some college or equivalent	32.5	31.3	33.6	38.3	37.1	39.5	42.0	40.1	43.9	32.4	31.5	33.2	36.3	35.2	37.4	41.7	40.8	42.5
Bachelor’s degree or higher	24.7	23.7	25.8	26.8	25.8	27.8	29.9	28.2	31.6	24.6	23.9	25.4	28.7	27.7	29.7	32.5	31.8	33.3
Don’t know	0.3	0.1	0.4	0.1	0.0	0.2	0.3	0.0	0.5	0.4	0.3	0.5	0.1	0.1	0.2	0.1	0.1	0.2
Refused to answer	0.6	0.3	0.8	0.2	0.1	0.3	0.2	0.0	0.4	0.7	0.6	0.9	0.2	0.1	0.3	0.3	0.2	0.4
**Ethnicity**																		
White	22.7	21.5	23.8	23.6	22.4	24.9	23.7	21.9	25.4	26.8	26.0	27.7	24.1	23.0	25.1	23.9	23.1	24.6
Other	77.3	76.2	78.5	76.4	75.1	77.6	76.3	74.6	78.1	73.2	72.3	74.0	75.9	74.9	77.0	76.1	75.4	76.9
**Total personal income**																		
Low	22.1	20.9	23.2	28.6	27.5	29.7	24.8	23.0	26.5	18.8	18.0	19.6	24.4	23.5	25.4	20.8	20.1	21.5
Low-average	26.2	25.0	27.4	28.3	27.2	29.4	25.6	23.9	27.3	27.9	27.0	28.8	31.1	30.1	32.2	29.8	29.0	30.6
High-average	20.3	19.2	21.4	19.4	18.4	20.3	19.3	17.9	20.8	20.9	20.1	21.7	20.2	19.3	21.1	19.8	19.2	20.5
High	20.7	19.7	21.8	17.5	16.6	18.4	22.8	21.2	24.4	21.4	20.6	22.1	18.6	17.6	19.5	22.3	21.6	23.0
Don’t know	2.5	2.1	3.0	2.5	2.1	2.8	2.7	2.0	3.4	3.4	3.0	3.8	2.4	2.0	2.7	2.5	2.3	2.8
Refused to answer	8.1	7.4	8.8	3.7	3.3	4.1	4.8	3.9	5.6	7.6	7.1	8.1	3.3	3.0	3.7	4.7	4.4	5.0
**Perception on the risk of daily cannabis vaping**																		
Low risk	14.6	13.6	15.6	20.5	19.4	21.5	19.1	17.6	20.6	11.5	10.8	12.1	19.9	19.0	20.9	18.6	17.9	19.2
Moderate risk	17.6	16.6	18.6	18.6	17.6	19.5	20.5	18.9	22.1	15.8	15.0	16.5	15.7	14.8	16.5	17.4	16.7	18.0
High risk	51.4	50.0	52.7	41.2	40.0	42.4	43.8	41.9	45.7	59.3	58.3	60.3	51.2	50.1	52.4	51.7	50.8	52.5
Don’t know	15.9	14.9	16.9	19.5	18.5	20.4	16.0	14.7	17.4	12.9	12.3	13.6	12.7	11.9	13.4	11.8	11.3	12.4
Refused to answer	0.5	0.3	0.8	0.3	0.2	0.4	0.6	0.3	0.9	0.5	0.4	0.7	0.5	0.3	0.7	0.6	0.4	0.7

## Appendix B

**Table A2:** Socio-demographics correlates and risk perception of vaping dried flower, oil or liquid, and concentrates in the past year, Canada ^a^.

	Vaping Dried Flower				Vaping Oil or Liquid				Vaping Concentrates				Any Vaping			
	OR	99.79% C.I		[*p*]	OR	99.79% C.I		[*p*]	OR	99.79% C.I		[*p*]	OR	99.79% C.I		[*p*]
**Age-group (ref: 56–65 years)**																
16–25 years	3.7 *	2.3	6.0	<0.001	4.5 *	3.0	6.8	<0.001	3.3 *	1.4	7.6	<0.001	3.9 *	2.8	5.6	<0.001
26–35 years	3.3 *	2.1	5.1	<0.001	3.9 *	2.6	5.7	<0.001	3.4 *	1.6	7.5	<0.001	3.6 *	2.6	4.9	<0.001
36–45 years	2.3 *	1.4	3.7	<0.001	3.0 *	2.0	4.4	<0.001	3.4 *	1.5	7.7	<0.001	2.5 *	1.8	3.6	<0.001
46–55 years	1.5	0.9	2.5	0.008	1.8 *	1.2	2.7	<0.001	1.7	0.7	3.9	0.051	1.6 *	1.1	2.3	<0.001
**Sex (ref: female)**																
Male	1.2	0.9	1.5	0.031	1.2	1.0	1.5	0.015	1.4	1.0	2.1	0.004	1.2 *	1.0	1.5	0.001
**Education level (ref: less than high school)**																
High school diploma or equivalent	1.0	0.6	1.7	0.962	1.2	0.8	1.8	0.241	1.4	0.7	3.0	0.167	1.2	0.8	1.7	0.157
Some college or equivalent	1.3	0.8	2.1	0.077	1.3	0.9	1.9	0.027	1.4	0.7	3.0	0.133	1.5 *	1.0	2.1	0.001
Bachelor’s degree or higher	1.3	0.8	2.2	0.078	1.0	0.7	1.5	0.966	0.8	0.3	1.8	0.397	1.2	0.8	1.7	0.219
**Ethnicity (ref: other)**																
White	1.1	0.8	1.5	0.276	0.9	0.7	1.2	0.219	1.0	0.6	1.5	0.950	1.0	0.8	1.3	0.791
**Total personal income (ref: low)**																
Low-average	1.1	0.7	1.7	0.447	1.2	0.9	1.7	0.074	1.6	0.8	2.9	0.024	1.2	0.9	1.6	0.034
High-average	1.4	0.9	2.2	0.014	1.3	0.9	1.9	0.015	1.5	0.8	3.0	0.048	1.3	1.0	1.8	0.006
High	1.6 *	1.0	2.5	0.002	1.6 *	1.1	2.3	<0.001	2.1 *	1.1	4.3	0.001	1.5 *	1.1	2.1	<0.001
**Perception on the risk of daily cannabis vaping (ref: high risk)**																
Low risk	8.6 *	6.2	12.1	<0.001	7.2 *	5.5	9.4	<0.001	7.5 *	4.4	12.7	<0.001	8.3 *	6.5	10.5	<0.001
Moderate risk	3.6 *	2.6	5.0	<0.001	3.6 *	2.8	4.8	<0.001	2.9 *	1.7	5.1	<0.001	3.7 *	2.9	4.6	<0.001

^a^ Each multivariable logistic regression model included survey year, age-group, sex, education level, ethnicity, total personal income, and perception on the risk of daily cannabis vaping as covariates. * aORs (adjusted odds ratios) were significant at the 0.002 level.

## Appendix C

**Table A3:** Socio-demographics correlates and risk perception of vaping dried flower, oil or liquid, and concentrates in the past year (U.S. illegal jurisdictions) ^a^.

	Vaping Dried Flower				Vaping Oil or Liquid				Vaping Concentrates				Any Vaping			
	OR	99.79% C.I		[*p*]	OR	99.79% C.I		[*p*]	OR	99.79% C.I		[*p*]	OR	99.79% C.I		[*p*]
**Age-group (ref: 56–65 years)**																
16–25 years	5.8 *	3.4	9.8	<0.001	3.5 *	2.5	5.0	<0.001	7.9 *	3.5	17.9	<0.001	4.0 *	2.8	5.5	<0.001
26–35 years	5.1 *	3.1	8.2	<0.001	3.5 *	2.6	4.9	<0.001	7.2 *	3.3	15.5	<0.001	4.0 *	2.9	5.4	<0.001
36–45 years	3.2 *	1.9	5.2	<0.001	2.3 *	1.7	3.3	<0.001	4.3 *	1.9	9.4	<0.001	2.5 *	1.9	3.5	<0.001
46–55 years	2.1 *	1.2	3.5	<0.001	1.5 *	1.1	2.2	<0.001	2.7 *	1.2	6.4	<0.001	1.6 *	1.1	2.2	<0.001
**Sex (ref: female)**																
Male	1.8 *	1.4	2.3	<0.001	1.5 *	1.3	1.9	<0.001	2.1 *	1.5	3.0	<0.001	1.6 *	1.4	1.9	<0.001
**Education level (ref: less than high school)**																
High school diploma or equivalent	1.3	0.8	2.2	0.073	1.4	1.0	2.0	0.007	1.8	0.8	4.1	0.035	1.4	1.0	2.0	0.003
Some college or equivalent	1.6	0.9	2.6	0.007	1.8 *	1.2	2.5	<0.001	1.9	0.8	4.4	0.029	1.8 *	1.2	2.5	<0.001
Bachelor’s degree or higher	1.5	0.9	2.5	0.027	1.5 *	1.0	2.2	0.002	1.7	0.7	4.3	0.065	1.5 *	1.0	2.1	0.001
**Ethnicity (ref: other)**																
White	0.9	0.6	1.2	0.218	1.2	0.9	1.5	0.075	1.2	0.8	2.0	0.187	1.0	0.8	1.3	0.660
**Total personal income (ref: low)**																
Low-average	1.2	0.8	1.7	0.209	1.1	0.8	1.4	0.498	1.0	0.5	1.7	0.863	1.1	0.8	1.4	0.282
High-average	1.5 *	1.0	2.3	0.001	1.0	0.8	1.4	0.839	1.4	0.8	2.6	0.064	1.2	0.9	1.5	0.117
High	2.1 *	1.4	3.2	<0.001	1.2	0.8	1.6	0.146	1.7	0.9	3.2	0.010	1.5 *	1.1	2.0	<0.001
**Perception on the risk of daily cannabis vaping (ref: high risk)**																
Low risk	5.1 *	3.7	6.9	<0.001	6.8 *	5.4	8.7	<0.001	6.1 *	3.8	10.0	<0.001	6.5 *	5.2	8.1	<0.001
Moderate risk	2.9 *	2.1	4.2	<0.001	3.2 *	2.5	4.2	<0.001	3.1 *	1.7	5.4	<0.001	3.2 *	2.5	4.1	<0.001

^a^ Each multivariable logistic regression model included survey year, age-group, sex, education level, ethnicity, total personal income, and perception on the risk of daily cannabis vaping as covariates. * aORs (adjusted odds ratios) were significant at the 0.002 level.

## Appendix D

**Table A4:** Socio-demographics correlates and risk perception of vaping dried flower, oil or liquid, and concentrates in the past year, U.S. legal jurisdictions ^a^.

	Vaping Dried Flower				Vaping Oil or Liquid				Vaping Concentrates				Any Vaping			
	OR	99.79% C.I		[*p*]	OR	99.79% C.I		[*p*]	OR	99.79% C.I		[*p*]	OR	99.79% C.I		[*p*]
**Age-group (ref: 56–65 years)**																
16–25 years	4.1 *	2.6	6.5	<0.001	2.9 *	2.2	3.8	<0.001	4.6 *	2.6	8.0	<0.001	3.3 *	2.5	4.2	<0.001
26–35 years	3.5 *	2.3	5.3	<0.001	3.1 *	2.4	3.9	<0.001	3.9 *	2.3	6.6	<0.001	3.2 *	2.5	4.0	<0.001
36–45 years	2.8 *	1.8	4.3	<0.001	2.6 *	2.0	3.4	<0.001	3.4 *	2.0	6.0	<0.001	2.6 *	2.1	3.4	<0.001
46–55 years	1.5	0.9	2.4	0.008	1.6 *	1.2	2.1	<0.001	1.8	1.0	3.2	0.003	1.6 *	1.3	2.1	<0.001
**Sex (ref: female)**																
Male	1.6 *	1.3	2.0	<0.001	1.2 *	1.0	1.4	0.001	1.6 *	1.2	2.0	<0.001	1.3 *	1.1	1.5	<0.001
**Education level (ref: less than high school)**																
High school diploma or equivalent	1.2	0.7	2.1	0.300	1.1	0.8	1.6	0.373	1.2	0.6	2.3	0.369	1.2	0.9	1.8	0.073
Some college or equivalent	1.4	0.8	2.4	0.054	1.2	0.8	1.7	0.119	1.6	0.9	2.9	0.018	1.3	0.9	1.9	0.010
Bachelor’s degree or higher	1.3	0.8	2.3	0.119	0.9	0.6	1.4	0.588	1.2	0.7	2.4	0.292	1.1	0.8	1.6	0.444
**Ethnicity (ref: other)**																
White	1.1	0.8	1.4	0.447	1.2 *	1.0	1.5	0.002	1.4 *	1.0	2.0	0.002	1.2 *	1.0	1.4	0.002
**Total personal income (ref: low)**																
Low-average	1.3	0.9	1.8	0.014	1.1	0.9	1.4	0.079	0.9	0.7	1.3	0.510	1.2	0.9	1.4	0.034
High-average	1.4	0.9	2.0	0.008	1.1	0.9	1.4	0.256	0.8	0.5	1.2	0.082	1.1	0.9	1.4	0.102
High	1.3	0.9	2.0	0.035	0.9	0.7	1.2	0.323	0.9	0.6	1.4	0.382	0.9	0.7	1.2	0.506
**Perception on the risk of daily cannabis vaping (ref: high risk)**																
Low risk	4.5 *	3.4	6.1	<0.001	7.0 *	5.8	8.5	<0.001	6.6 *	4.7	9.3	<0.001	7.0 *	5.8	8.4	<0.001
Moderate risk	2.6 *	1.9	3.6	<0.001	3.9 *	3.1	4.7	<0.001	3.5 *	2.4	5.2	<0.001	3.6 *	3.0	4.4	<0.001

^a^ Each multivariable logistic regression model included survey year, age-group, sex, education level, ethnicity, total personal income, and perception on the risk of daily cannabis vaping as covariates. * aORs (adjusted odds ratios) were significant at the 0.002 level.
